# Neuronal Cell Differentiation of iPSCs for the Clinical Treatment of Neurological Diseases

**DOI:** 10.3390/biomedicines12061350

**Published:** 2024-06-18

**Authors:** Dong-Hun Lee, Eun Chae Lee, Ji young Lee, Man Ryul Lee, Jae-won Shim, Jae Sang Oh

**Affiliations:** 1 Industry-Academic Cooperation Foundation, The Catholic University of Korea, 222, Banpo-daro, Seocho-gu, Seoul 06591, Republic of Korea; 2 Department of Medical Life Sciences, College of Medicine, The Catholic University of Korea, Seoul 06591, Republic of Korea; 3 Department of Neurosurgery, Uijeongbu St. Mary’s Hospital, College of Medicine, The Catholic University of Korea, Seoul 06591, Republic of Korea; 4 Soonchunhyang Institute of Medi-Bio Science (SIMS), Soonchunhyang University, Cheonan-si 31151, Republic of Korea; 5 Department of Integrated Biomedical Science, Soonchunhyang University, Cheonan-si 31151, Republic of Korea

**Keywords:** BMP-4 protein, induced pluripotent stem cells, neural stem cells, SMAD proteins, transforming growth factor beta, transplantation

## Abstract

Current chemical treatments for cerebrovascular disease and neurological disorders have limited efficacy in tissue repair and functional restoration. Induced pluripotent stem cells (iPSCs) present a promising avenue in regenerative medicine for addressing neurological conditions. iPSCs, which are capable of reprogramming adult cells to regain pluripotency, offer the potential for patient-specific, personalized therapies. The modulation of molecular mechanisms through specific growth factor inhibition and signaling pathways can direct iPSCs’ differentiation into neural stem cells (NSCs). These include employing bone morphogenetic protein-4 (*BMP-4*), transforming growth factor-beta (*TGFβ*), and Sma-and Mad-related protein (SMAD) signaling. iPSC-derived NSCs can subsequently differentiate into various neuron types, each performing distinct functions. Cell transplantation underscores the potential of iPSC-derived NSCs to treat neurodegenerative diseases such as Parkinson’s disease and points to future research directions for optimizing differentiation protocols and enhancing clinical applications.

## 1. Introduction

Neurological disorders, especially cerebrovascular diseases and strokes, are a significant global issue [1]. These conditions lead to irreversible neural damage, and currently, there are limited effective treatments available for repairing damaged tissue or restoring function [2,3,4]. To overcome this, regenerative medicine has begun to focus on the differentiation of neural cells from induced pluripotent stem cells (iPSCs) [5].

Stem cells inherently possess two key functions: the capacity for unlimited self-renewal and the ability to differentiate into one or more specialized cell types [6]. These characteristics play a fundamental role in exploring tissue repair and disease treatment methods through stem cells [7].

iPSCs are cells that have regained pluripotency through the reprogramming of already differentiated mature cells and are created by manipulating the expression of specific genes [8,9]. The technology of iPSCs, which restores pluripotency from mature cells, offers innovative potential for generating patient-specific disease models and developing personalized treatments [10]. Neural cells generated from iPSCs can be used to replace or repair damaged neural tissue [11]. Moreover, using neural cells differentiated from patient-derived iPSCs allows for effective testing of new drugs’ efficacy or toxicity [12]. Transplanting these iPSC-derived neural cells could lead to functional recovery in neurodegenerative diseases such as Alzheimer’s or Parkinson’s disease.

Against this background, it is expected that the process of neuronal differentiation of iPSCs will be examined, and the mechanisms of neuronal differentiation will be elucidated, providing an important step in the development of regenerative medicine and disease therapies.

## 2. Inhibiting the SMAD Pathway in iPSCs for Neural Differentiation

The process of differentiating iPSCs into various cells includes several complex signaling pathways and molecular mechanisms. iPSCs have important advantages over embryonic stem cells (ESCs). iPSCs are derived from adult cells; they bypass the ethical issues of destroying embryos to derive ESCs [13,14]. iPSCs can be self-derived from the patient, allowing for the creation of patient-specific cell lines [12,15]. They can differentiate into multiple cell types, allowing drug testing to assess effectiveness and identify side effects safely and efficiently [16]. Furthermore, iPSCs retain the same pluripotency as that of ESCs [17]. Both iPSCs and ESCs exhibited equivalent neuronal differentiation potential, and both cells showed similar cholinergic motor neuron differentiation potential and the ability to induce the contraction of myotubes [18]. In another study, while iPSC-derived neural stem cells (NSCs) had decreased ATP production compared to that of ESC-derived NSCs, iPSC-derived astrocytes had increased ATP production compared to that of ESC-derived astrocytes [19].

**Figure 1:**
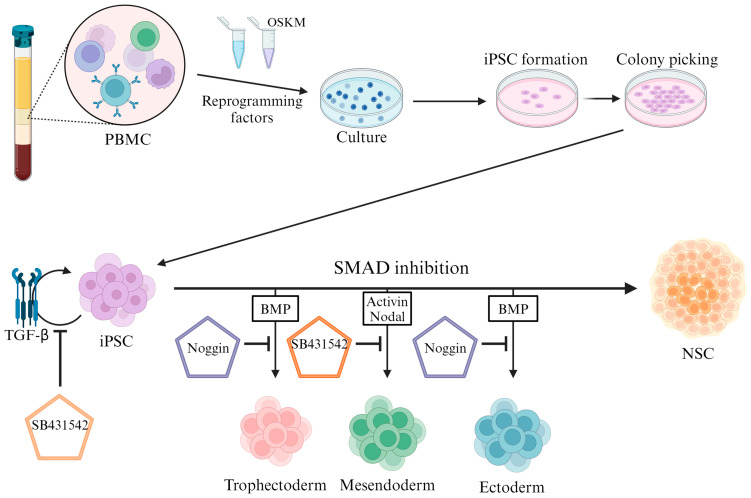
Adding reprogramming factors to PBMCs to induce their reverse differentiation into iPSCs. Reverse-differentiated iPSCs can be induced to undergo mesoderm or endoderm differentiation through the activation of the SMAD pathway. Inhibition of the SMAD pathway induces the neural stem cell differentiation of iPSCs. *BMP*: bone morphogenetic protein, *TGFβ*: transforming growth factor-beta, NSC: neural stem cell, iPSC: induced pluripotent stem cell, PBMC: peripheral blood mononuclear cell, OSKM: *Oct4*/*Sox2*/*Klf4*/*c-Myc*, SMAD: *Sma*- and *Mad*-related protein.

Specifically, the differentiation of neuronal cells is induced by the dual inhibition of the Sma- and Mad-related protein (SMAD) pathway (Figure 1). Before understanding the SMAD pathway, it is necessary to understand the transforming growth factor-beta (*TGFβ*) signaling pathway, which includes SMAD.

### 2.1. SMAD Pathway Inhibition

Inhibition of the SMAD pathway directs the fate of iPSCs towards the neuroectoderm and induces neural cell differentiation through the inhibition of *TGFβ* and *BMP-4* signaling, as mentioned above [20]. For the dual inhibition of the SMAD pathway, SB431542 is used to inhibit the *TGFβ* pathway and *Noggin* is used to inhibit the *BMP* pathway.

SB431542 inhibits the *Lefty*/*Activin*/*TGFβ* pathway by blocking the phosphorylation of *ALK4*, *ALK5*, and *ALK7* receptors. SB431542 also inhibits differentiation to the mesoderm by inhibiting *Activin*/*Nodal* signaling. *Noggin* inhibits differentiation to the ectoderm by inhibiting the *BMP* pathway. A combined treatment of SB431542 and *Noggin* induced the neural differentiation of stem cells with high efficiency [20]. The mechanisms by which *Noggin* and SB431542 induced neural cell differentiation include *Activin*- and *Nanog*-mediated network destabilization [21], *BMP*-induced inhibition of differentiation [22], and the inhibition of mesodermal and endodermal differentiation through the inhibition of endogenous *Activin* and *BMP* signaling [23,24]. Treatment with SB431542 decreases *Nanog* expression and significantly increases *CDX2* expression. The inhibition of *CDX2* in the presence of *Noggin* or SB431542 demonstrates that one of the key roles of *Noggin* is the inhibition of endogenous *BMP* signaling, which induces trophoblast fate during differentiation.

### 2.2. TGFβ Signaling Pathway

The *TGFβ* signaling pathway is a pathway that regulates cell growth, differentiation, migration, death, and homeostasis [25]. The superfamily of *TGFβ* includes bone morphogenetic protein (*BMP*), *Activin*, *Nodal*, and *TGFβ*. Signal transduction in this pathway begins with the binding of superfamily ligands of *TGFβ* to *TGFβ* receptor type II and *TGFβ* receptor type I [26]. Activated *TGFβ* receptors recruit *Smad2/3* for *TGFβ* and activation signaling [27] and form complexes of CoSmad and R-smad, such as *Smad4*, for *BMP* signaling [28]. Smad complexes accumulate in the nucleus and are directly involved in the transcriptional regulation of target genes [29].

### 2.3. BMP Signaling Pathway

*BMP*s are cytokines that belong to a group of growth factors [30]. *BMP*s have a role in early skeletal formation during embryonic development and were originally known to act as bone growth factors [31]. *BMP*s bind to a heteromeric receptor complex composed of type I and type II serine/threonine kinase receptors, which are received by different activin receptors and *BMP* receptors [32]. The two receptors are highly homologous and can activate both Smad and non-Smad signaling.

*BMP-4* is a member of the *BMP* superfamily, which induces the ventral mesoderm to establish dorsal–ventral morphogenesis. *BMP4* signaling is found in the formation of early mesoderm and germ cells, and the development of the lungs and liver is attributed to *BMP4* signaling [33]. Inhibition of this *BMP-4* signaling induces neurogenesis and the formation of the neural plate. Indeed, the knockout of *BMP-4* in mice resulted in little mesodermal differentiation [34].

### 2.4. RA Pathway

Retinoic acid (RA) is a molecule that contributes to the development and homeostasis of the nervous system [35]. The RA signaling depends on cells having the ability to metabolize retinol. Transcription is regulated by the binding of RA to its receptor, RA receptor (*RAR*), which forms a complex with the retinoid X receptor (*RXR*) [36]. The RA is involved in the differentiation of NSCs into neurons, astrocytes, or oligodendrocytes [37]. RA activates the *Hox* gene, which is required for hindbrain development and regulates the head–trunk transition [38]. RA is required for the formation of primary neurons [39]. In an embryonal carcinoma cell line in vitro, RA promoted neurite outgrowth and stimulated the expression of neural differentiation markers [40].

Furthermore, RA is essential in embryonic development and is essential for the development of many organs, including the hindbrain, spinal cord, skeleton, heart, and brain [41].

### 2.5. BDNF, GDNF, and NGF Pathway Regulation

Brain-derived neurotrophic factor (BDNF) is a neurotrophic factor found primarily in the brain and central nervous system that regulates nerve cell survival, growth, and neurotransmission [42]. BDNF promotes neuronal survival and growth in dorsal root ganglion cells and in hippocampal and cortical neurons [43,44]. In in vitro experiments in which neural differentiation was induced in a variety of stem cells, neural differentiation was confirmed after treatment with BDNF [45,46].

Glial-cell-line-derived neurotrophic factor (GDNF) is a protein that promotes the survival of many different neurons [47]. GDNF can be secreted by neurons and peripheral cells during development, including astrocytes, and interacts with GDNF family receptor alpha 1 and 2 [48]. In particular, it has a protective effect on dopamine-producing nerve cells, making it an important target in neurodegenerative diseases such as Parkinson’s disease [49].

Nerve growth factor (NGF) is a neuropeptide involved in regulating the growth, proliferation, and survival of neurons [50]. In in vivo and in vitro studies, NGF has been shown to have an important role in the differentiation and survival of neurons, as well as in the protection of degenerating neurons.

## 3. Differentiation of Various Neural Cells from iPSCs

**Figure 2:**
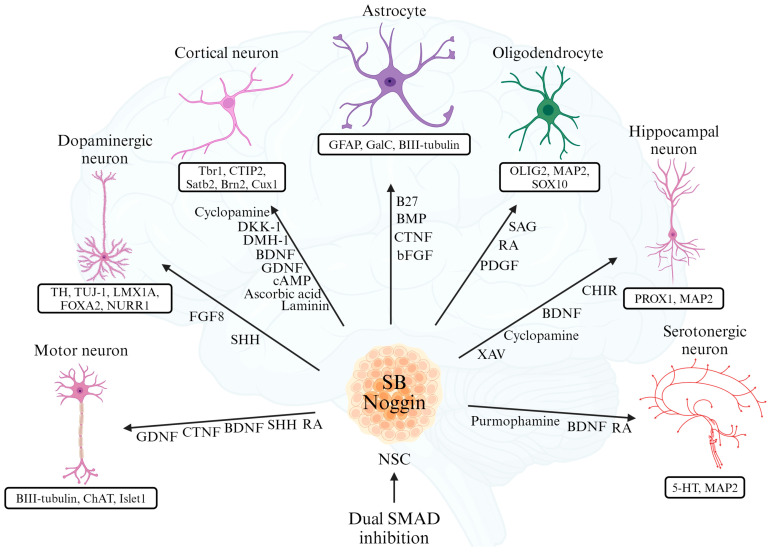
Different neural cells that can differentiate from neural stem cells. Cells induced to become neural stem cells due to the inhibition of the dual SMAD pathway with SB431542 and *Noggin* can be combined to add cytokines specific to each differentiation target. The cytokines described next to the black arrows indicate the fate of each neuron. Differentiated neurons are identified through the detection of the proteins listed under each neuron.

Through various mechanisms, neural cell differentiation from iPSCs can develop a diverse array of neurons (Figure 2, Table 1). It is possible to consider prior studies that successfully differentiated various neurons from iPSCs and the application of protocols used for the differentiation of human ESCs (hESCs) into iPSCs.

### 3.1. Differentiation into Cortical Neurons

iPSCs can differentiate into cortex neurons. The study by Kaveena Autar [51] induced an initial neural lineage in iPSCs using two small molecule inhibitors of the SMAD pathway, LDN193189 and SB431542, promoting neuroepithelial differentiation. Following the early neural induction, the neural epithelium was induced using *DKK-1*, a *Wnt/B* antagonist, and *DMH-1*, a *BMP* inhibitor, enhancing the development of rostral neuroepithelial cells. Finally, the application of cyclopamine, an *SHH* inhibitor, designated the cortex fate, while BDNF, GDNF, cAMP, ascorbic acid, and laminin improved the generation of cortical neurons.

In the research by Yichen Shi, cortical development was induced in both hESCs and iPSCs using dorsomorphin, an inhibitor of the SMAD pathway [52].

Cortical differentiation can be confirmed by the reduced expression of the pluripotency gene *Oct4* and the increased expression of the genes *Tbr1*, *CTIP2*, *Satb2*, *Brn2*, and *Cux1*.

### 3.2. Differentiation into Dopaminergic Neurons

Human iPSCs are capable of differentiating into midbrain dopaminergic neurons. In a study by Lixiang Ma, dopaminergic neurons were generated from iPSCs [53]. After inducing iPSCs into neural epithelial cells, applying *FGF8* and *SHH* efficiently produced dopaminergic neurons from midbrain precursors without the need for co-culture. Dopaminergic neurons can be identified by detecting markers such as *TH*, *TUJ-1*, *LMX1A*, *FOXA2*, and *NURR1*.

It is also possible to induce the dopaminergic neuronal differentiation of iPSCs without the use of pharmacological compounds for the inhibition of SMAD mechanisms [54]. Adeno-associated viral vectors were designed to upregulate *Lmx1a* through *SHH* and *Wnt* and then transfected into iPSCs. The iPSCs not only successfully generated dopaminergic neurons but also showed a consistent number of them.

### 3.3. Differentiation into Motor Neurons

iPSCs can differentiate into motor neurons [55]. After inducing iPSCs into embryonic bodies, treatment with RA and purmorphamine, an activator of the sonic hedgehog pathway, resulted in the expression of neural precursor markers. Cells forming neural rosettes were mechanically separated, plated in media containing RA and *Shh*, and cultured for a week. Following further culture with BDNF, CTNF, GDNF, and *Shh*, after 3–5 weeks, cells displayed motor neuron characteristics, and *BIII-tubulin*, *ChAT*, and *Islet1* were detected.

### 3.4. Differentiation into Astrocytes

iPSCs can differentiate into astrocytes [56]. iPSCs induced into NSCs were cultured in NSC media containing B27, *BMP*, CTNF, and *bFGF*. The differentiated astrocytes were co-cultured with the neuron layer. Throughout the culture, neurons were distinguished by their distinct cell bodies and measured along axons using fluorescence imaging. Neurons and astrocytes, as well as oligodendrocytes, were differentiated by expressing markers such as *BIII-tubulin*, *GFAP*, and *GalC*.

### 3.5. Differentiation into Oligodendrocytes

iPSCs can differentiate into oligodendrocytes [57]. Neural differentiation was induced through dual SMAD inhibition. After differentiation, adding *SAG* and RA promoted sphere aggregation, and using PDGF media encouraged OPC formation. The development of oligodendrocytes was confirmed through the detection of *OLIG2*, *MAP2*, and *SOX10*.

### 3.6. Differentiation into Hippocampal Neurons

NSCs derived from iPSCs can differentiate into the hippocampus [58]. Neural induction media composed of B27, N2, and *NEAA* were supplemented with LDN-193189, Cyclopamine, SB431542, and XAV-939 to induce differentiation, and CHIR-99021 and BDNF were added to promote hippocampal neuron development. The generation of hippocampal neurons was confirmed through the detection of *PROX1*.

### 3.7. Differentiation into Serotonergic Neurons

NSCs derived from iPSCs can differentiate into serotonergic neurons [59]. Human pluripotent stem cells (hPSCs) were cultured in an N2 medium combined with a knockout serum replacement medium and treated with SB431542, LDN193189, purmorphamine, and RA. After 11 days, the medium was switched to NB/B27 medium, and BDNF was added. Following differentiation, the presence of serotonergic neurons was confirmed through immunofluorescence staining for 5-HT, *MAP2*, *TUJ1*, *FEV*, and *TPH2* expression. Subsequent 3D culture also successfully yielded organoids, and the release of *5-HT* and its metabolites was observed.

## 4. Therapeutic Research Using Neural Cells Derived from iPSCs

Researchers are hopeful that the transplantation of neural cells derived from iPSCs can overcome neurodegenerative diseases. To treat Parkinson’s disease, which has been identified as a disorder of dopaminergic neurons, the transplantation of iPSC-derived dopaminergic neurons is considered. If these transplanted neurons function normally, they could potentially cure Parkinson’s disease. This anticipation has led to the execution of cell transplantation therapies targeting either cells or animals, and in some cases, applications have extended to clinical trials.

### 4.1. Dopaminergic Neuron Therapy in a Model of Parkinson’s Disease 

Dopaminergic neurons from PSCs may be a candidate for the treatment of Parkinson’s disease. When dopaminergic neurons were transplanted into the nigrostriatal lesions of rats with Parkinson’s disease, the neurons survived and interacted in the rats’ brains for a long period of time [60]. After cell transplantation, the rats’ motor function was restored.

### 4.2. In Vivo Transplantation and Survival of Astrocytes

Astrocytes derived from PSCs were transplanted into the striatum of mice to investigate their survival and function [56]. In the brains of mice obtained 2 weeks after astrocyte transplantation, *GFAP*-positive cells were still observed.

Furthermore, when iPSC-derived astrocyte progenitors were transplanted into the brain of an Alzheimer’s disease model in mice and examined through immunostaining, they interacted and functionally integrated with other cells in vivo [61].

### 4.3. Survival of Oligodendrocytes after Transplantation in Mice

To investigate the function of iPSC-derived oligodendrocytes, cells were injected into the forebrain of immunocompromised mice. At 12 weeks after cell injection, the oligodendrocytes were detected through immunofluorescence staining of hNA+ and *OLIG2* protein in the corpus callosum.

### 4.4. Clinical Trials with iPSC Transplantation

There are very few studies in which iPSCs have been transplanted into humans. This is because questions about the safety, stability, and efficacy of iPSCs are constantly being raised. The first thing that researchers worry about is the ability to form tumors, which is a common concern in stem cell research [62]. iPSCs also have a theoretical risk of forming tumors, so safety considerations follow. In addition, treatments using iPSC technology may result in modifications to the human genome, which requires discussion of the long-term ethical implications. For example, concerns include human cloning or human–animal chimeras.

On the other side of the spectrum, there are also concerns related to the immune response. Even though iPSCs are self-derived cells, the immune system may recognize them as foreign and attack them [63,64]. This can happen mainly due to mismatches in human leukocyte antigens (HLAs), which is why it is important to select cells based on HLA matching. If iPSCs are generated from a donor with a specific HLA type, it is possible to use iPSCs from other people [63]. If an HLA is incompatible, one can also modulate HLA expression or use gene editing [64].

Finally, because iPSCs must undergo reverse differentiation from human-derived cells, it takes a significant amount of time just to generate the cells. This can make it difficult to use autologous cells to treat acute illnesses.

In 2020, a transplantation study of iPSC-derived dopamine progenitor cells for the treatment of Parkinson’s disease patients was conducted [65]. After harvesting fibroblasts by skin biopsy, dopamine progenitor cells were characterized in vitro with dopamine-neuron-specific and other neuronal markers. Characterized dopamine progenitor cells were transplanted into patients with Parkinson’s disease, and Parkinson’s-disease-related measures were assessed at 1, 3, 6, 9, and 12 months and every 6 months thereafter. Transplanted cells survived for 2 years without side effects. F-DOPA PET-CT imaging from 0 to 24 months showed a modest increase in dopamine uptake in the posterior cingulate near the implantation site. They also showed improved quality of life in clinical assessments of motor signs in Parkinson’s disease, although interpretation should be carried out with caution due to the lack of a control group comparison.

In 2021, there was a planned clinical study of the transplantation of iPSC-derived neural progenitor cells for the treatment of subacute complete spinal cord injury [66]. However, this was postponed due to the sudden onset of the COVID-19 pandemic. A clinical-grade iPSC line (YZWJs513) prepared at the GMP facility of Osaka National Hospital was induced to differentiate into neural progenitor cells (NPCs), and preclinical studies using mouse models confirmed its promotion of motor function recovery after spinal cord injury.

## 5. Conclusions

iPSCs can differentiate into a variety of neuronal cell types, including dopaminergic neurons, astrocytes, and microglia, which could be a revolutionary way to treat a variety of neurodegenerative diseases. Inhibition of TGFβ and the SMAD pathway induces neural progenitor cell differentiation of cells with restored pluripotency. The differentiated cells still survive and function in the body.

The chemical drugs used to treat neurodegenerative diseases have different susceptibilities in different patients and have short half-lives, meaning that they are quickly used up by the body. Drugs for neurodegenerative diseases such as Parkinson’s disease and Alzheimer’s disease can slow their progression by increasing the release of neurotransmitters, but they cannot reverse the course of the disease. In addition, unlike a body part such as an arm, it is very difficult to accurately deliver chemical drugs to the brain. Cell transplantation treatments using patient-derived iPSCs are entirely patient-derived, have a high degree of tolerance, and may be able to survive and function in the long term to reverse the progression of neurodegenerative diseases.

However, clinical experimental studies of iPSCs and neural progenitor cells differentiated from them are extremely rare and require careful handling. The response in experimental animals and humans may be different, and we do not yet fully understand the differentiation of iPSCs.

Future research should focus on optimizing protocols for iPSC-derived neural cell differentiation, ensuring long-term viability and the functional integration of transplanted cells in vivo and paving the way for clinical applications.

## Figures and Tables

**Table 1:** Strategies for iPSCs differentiated into neural progenitor cells to become multifunctional neurons.

References	Type of Neuron	Differentiation Inducers	Specific Markers
[51,52]	Cortical Neurons	Cyclopamine, DKK-1, DMH-1, BDNF, GDNF, cAMP, Ascorbic acid, Laminin	Tbr1, CTIP2, Satb2, Brn2, Cux1
[53,54]	Dopaminergic Neurons	FGF8, SHH	TH, TUJ-1, LMX1A, FOXA2, NURR1
[55]	Motor Neurons	GDNF, CTNF, BDNF, SHH, RA	BIII-tubulin, ChAT, Islet1
[56]	Astrocytes	B27, BMP, CTNF, bFGF	GFAP, GalC, BIII-tubulin
[57]	Oligodendrocytes	PDGF, RA, SAG	OLIG2, MAP2, SOX10
[58]	Hippocampal Neurons	CHIR, BDNF, Cyclopamine, XAV	PROX1, MAP2
[59]	Serotonergic Neurons	Purmophamine, BDNF, RA	5-HT, MAP2

## Abbreviations

BDNF	Brain-derived neurotrophic factor
BMP	Bone morphogenetic protein
ESC	Embryonic stem cell
GDNF	Glial-cell-line-derived neurotrophic factor
HLA	Human leukocyte antigen
iPSC	Induced pluripotent stem cell
NPC	Neural progenitor cell
NSC	Neural stem cell
PSC	Pluripotent stem cell
RA	Retinoic acid
RAR	Retinoic acid receptor
RXR	Retinoid X receptor
SMAD	Sma- and Mad-related protein
TGFβ	Transforming growth factor-beta
